# Dynamic Computational Model Suggests That Cellular Citizenship Is Fundamental for Selective Tumor Apoptosis

**DOI:** 10.1371/journal.pone.0010637

**Published:** 2010-05-13

**Authors:** Megan Olsen, Nava Siegelmann-Danieli, Hava T. Siegelmann

**Affiliations:** 1 Department of Computer Science, University of Massachusetts Amherst, Amherst, Massachusetts, United States of America; 2 CommuniSef.com and Maccabi Health Services, Tel-Aviv, Israel; Tel Aviv University, Israel

## Abstract

Computational models in the field of cancer research have focused primarily on estimates of biological events based on laboratory generated data. We introduce a novel in-silico technology that takes us to the next level of prediction models and facilitates innovative solutions through the mathematical system. The model's building blocks are cells defined phenotypically as normal or tumor, with biological processes translated into equations describing the life protocols of the cells in a quantitative and stochastic manner. The essentials of communication in a society composed of normal and tumor cells are explored to reveal “protocols” for selective tumor eradication. Results consistently identify “citizenship properties” among cells that are essential for the induction of healing processes in a healthy system invaded by cancer. These properties act via inter-cellular communication protocols that can be optimized to induce tumor eradication along with system recovery. Within the computational systems, the protocols universally succeed in removing a wide variety of tumors defined by proliferation rates, initial volumes, and apoptosis resistant phenotypes; they show high adaptability for biological details and allow incorporation of population heterogeneity. These protocols work as long as at least 32% of cells obey extra-cellular commands and at least 28% of cancer cells report their deaths. This low percentage implies that the protocols are resilient to the suboptimal situations often seen in biological systems. We conclude that our in-silico model is a powerful tool to investigate, to propose, and to exercise logical anti-cancer solutions. Functional results should be confirmed in a biological system and molecular findings should be loaded into the computational model for the next level of directed experiments.

## Introduction

Cancer incidence is expected to rise worldwide from 12 million new people affected annually in the year 2000 to an anticipated 20 million in the year 2030, highlighting the urgent need to identify highly effective preventative and therapeutic interventions. This paper introduces an original mathematical system, combining Dynamical Systems theory and Artificial Intelligence algorithms in an attempt to identify logical principles underlying cancer development and imply innovative anti-cancer solutions. The model simplifies the complex environment of cancer development and progression, where numerous chemical, biological, and physical factors act together to affect intra-cellular events and extra-cellular signaling. While simplification is essential to unmask the fundamental principles of cancer occurrence, the artificial intelligence component of our system affords a high level of adaptation for numerous intra- and extra-cellular details, unlike previous cancer models that were restricted to probabilities of several intra-cellular events [1]–[8]. Our model provides a framework to assess several important questions in Oncology: What kind of information flow inside and between cells may be associated with tumor development and progression; What kind of inter-cellular communication keeps tumor cells dormant; Do current therapies bias some of the natural flow to explain their temporary benefit; And what are the principles of successful inter-cellular communication rules that would enable selective tumor cells' apoptosis (programmed cell death). The latter subject is the focus of this manuscript.

Three assumptions are made within the model [9]. First, it is assumed that intra-cellular biological cascades and extra-cellular signaling can be measured by units and be quantified by mathematical equations; this relates to Information Substance Theory where biological events are interpreted as flow of information units [10]. Second, natural mechanisms for selective cancer cell death in living organisms are assumed to exist, as otherwise cancer incidence as calculated by mutation rates would be significantly higher [2]. Analogous mechanisms have been reported and include tumor removal via immune surveillance [11].

Third, we relate two seemingly opposing biological facts about cancer and apoptosis: The classic hallmark of cancer, that cancer arises when inappropriate apoptotic response occurs and prevents natural eradication of mutated cells [12], and the induction of caspace-dependent tumor cell apoptosis as a universal mechanism for tumor cell death by irradiation and the majority of chemotherapy agents [13]–[15]. This leads to our third assumption that even highly mutated cells maintain residual apoptotic abilities that we name “basic citizenship responsibilities.” We further assume that those skills require communication with other cells in the system, both by signal emission on viability status (alive or dead) and the compliance with external apoptotic commands. These assumptions are supported by mounting evidence on the significance of the tumor environment in cancer occurrence, with data from several studies pointing directly to our assumptions. There are data on the role of high mobility group box 1 (HMBOX1) protein in reporting cancer cell death to the immune system as an essential part of tumor death by chemotherapy agents [16], and findings showing that AP2L/TRAIL (tumor-necrosis-factor-related apoptosis induced ligand) binding to “death receptors” DR4 and DR5 induces selective tumor cell apoptosis via the external apoptosis pathway [17]–[19].

Our model seeks to provide a single unifying mechanism incorporating these assumptions [16]–[19]. It is a dynamic tissue simulation model composed of cells cycling in a 3-dimensional society, where normal and mutated cells are defined phenotypically by their apoptotic response, proliferation pace, and compliance with spatial regulation rules. Signaling toward apoptosis is modeled in terms of the flow of information that activates the intrinsic and extrinsic paths correlating with activities of intrinsic regulating factors, and extrinsic modifiers, respectively [17], [18]. Findings suggest that our modeling may constitute a complementary approach to biological research; directions proposed by the self learning model (upon incorporation of resultant biological details and a definition of optimal results) could lead to an enhanced understanding of these processes and of potential interventions.

## Methods

**Figure 1:**
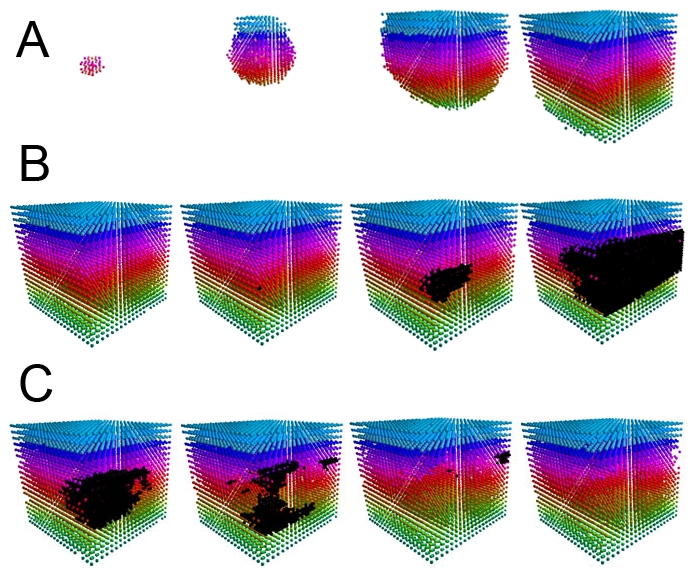
The progression of a simulation. Color is utilized for visualization purposes, with black representing tumor cells. (a) The initial system growth starting with a single cell in increments of 50 steps. (b) The growth of a tumor, starting from a single cell, in increments of 10 computational time steps. (c) The rescue protocols working to remove the tumor cells without significant damage to the surrounding tissue at time steps 18, 20, and 25 after tumor planting.

The system is written in the object-oriented C++ programming language, and is initiated as a single cell that via replication and structural regulations fills a given size 3-dimensional membrane (Figure 1a). Once the membrane is filled, the system maintains its structural and functional homeostasis. Simulated cells are regulated by basic life protocols that sense and affect both their internal states and their environment, and jointly support the system's goals: Spatial regulations (contact inhibition), control of individual cell health via repair of mutations, and system longevity and integrity.

Models using various levels of detail exist, all aimed at simplifying the complexities of cancer occurrence in order to reveal fundamental biological processes. Choosing which details to remove and include should be suggested by the specific goal being investigated. In our case, the goal is to determine core information about the types of “rescue protocols” internal to the cells, which are an essential part of the ability to fight the growth of mutated cells and prevent them from overwhelming the system. Consequently, we select the details we will focus on based on the necessities for modeling this type of occurrence, and generalize the rest.

### The Life Protocols

The **basic life protocols** reflect **proliferation** (including rate parameters, generation potential, and space restrictions [9], [20]–[22]), **proliferation-suppression** mechanisms, **self-testing** at a check point prior to the replication decision, **repairing** damages, and **apoptosis**. The latter is activated as either a random process, secondary to a cell's decision to die due to aging or uncorrected defects, or as a reaction to extra-cellular signaling. The **distance regulation** **protocol** maintains shape cohesiveness and allows undisturbed communication flow among cells [8]. These protocols are chosen to approximate the healthy functioning of phenotypic properties of cycling cells. The physical property of space is important in our model since normal cells proliferate only when given space around them, whereas tumor cells may violate this restriction. This can be a basis for the creation of solid tumors of a particular shape, as our cells grow in an expected spheroid pattern with current spatial parameters even though we do not explicitly model this behavior [23], [24].

Tumor cells can develop in the system spontaneously (see results) from a cell whose life protocols are damaged; however, to speed up and ease the analysis of growth after the initial tumor cell develops, we can also plant a single cancer cell into the model at a set time for every experiment. A tumor cell in our model, once created, can only produce tumor cells and cannot back mutate into a normal cell.

Three techniques are used to provide biological plausibility and applicability to the simulation results. They are population heterogeneity, a relative proliferation ratio, and varied experimentation.

#### Population Heterogeneity

Variances are created by ranges of death and proliferation probabilities within each cell population, which vary by +/−200% and +/−300% respectively. A newly created daughter cell inherits these probabilities with random skewing from the parent's characteristics. To assure probabilistic cover for feasible ranges of variables due to the difficulty of estimation, we include large ranges of values.

#### Relative proliferation ratio

This factor describes the quotient of the valid range of tumor and normal proliferation probabilities. For demonstrations we use ratios of 6, 10, and 20. This ratio characterizes the tumor cells in terms of how much their proliferation is increased over the normal cells. For each ratio the tumor proliferation rate is taken to be in the range of 0.3 to 0.9, and the tumor death rate is 0.0001 to 0.0002. The normal proliferation rates are in the range of 0.05 to 0.15 for ratio 6, in 0.03 to 0.09 for ratio 10, and in 0.015 to 0.045 for ratio 20. The normal death rates are within the wide range of 0.0024 to 0.0048 for all ratios. These ranges are based on biological data [25].

#### Varied Experimentation

To ensure more accurate results, each set of parameters is tested multiple times since even for a fixed set of parameters describing the system the end result may differ as life protocols of individual cells are defined in a stochastic manner. This increases the realism of results, as it ensures that we test many different possible outcomes from the same set of basic parameters in the system.

### Anti-Cancer Interventions

The major innovation of this research is discovering potentially natural “rescue protocols” that have the ability to prevent many occurrences of evolved cancer cells from overrunning the system. These protocols are activated once the system detects a risk to its existence. Successful protocol activation is defined as having all cancer cells die at the end of the process without irreversibly wiping out the healthy system.

Numerous hypothetical protocols are tested by varying, for example, the type of cells initiating signals (normal, tumor, partially mutated cells), the conditions causing signal initiation (e.g., spatial violation), and emitted signal properties and parameters (strength, radius).

## Results

### Tumor Developmental Model

A tumor cell originates through mutations to basic life protocols of a normal cell, and may occur only through particular orderings of mutations that bypass normal guarding mechanisms: repair, apoptosis, proliferation-suppression, and distance regulation. Tumor cells may over-replicate to form tumor clusters that blindly occupy space, creating pressure on nearby healthy tissue (Figure 1b). This pressure suppresses normal cells' proliferation rates due to the distance regulation protocol representing contact inhibition. This further increases the gap between proliferation activities of normal and mutated cells.

### The Rescue Protocols

**Figure 2:**
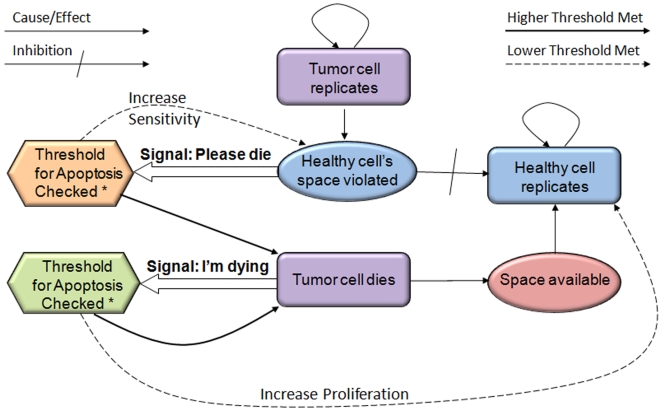
The “I'm Dying” is necessary to complement the “Please Die” protocol. The closed loop of tumor cells' death biases the system toward killing tumor cells at internal parts of the cancer cluster and promotes normal cell repopulation.

From in-silico experiments comparing numerous communication rescue protocols (tens of thousands of runs), the following combined protocol was determined to show the optimal outcome (Figure 2):

1. **Please Die Protocol**. Normal cells initiate signals called “please die” when they are spatially (physically) violated by neighboring tumor clusters. Receiving cells consider the total strength of signals received over time. If the “higher threshold” is reached in a cell it will induce apoptosis. A “lower threshold” is also defined for normal cells only; in response to it they will emit “please die” signals sooner, causing resistance to tumor re-growth.
2. **I'm Dying Protocol**. Cells dying due to either the “please die” or the “I'm dying” protocol will emit “I'm dying” signals. This protocol is a basic cellular “citizenship” commitment. Receiving cells consider the total strength of signals received and act in accordance with one of two thresholds: The higher threshold provokes apoptosis and due to spatial regulations affects mostly internal tumor parts; the lower threshold accelerates normal cells' replication rate to promote repopulation of evacuated space. This process is inspired by similar phenomena described in Drosophila which is mediated via JNK and the Wingless signaling pathways [21].

The “please die” protocol can only cause removal of mutated cells from the tumor periphery, which is not sufficiently realistic. The signal “I'm dying” transfers the death messages deeper into the tumor cluster (Figure 2). The combined protocol follows cancer growth dynamically in real-time and is typically victorious in its anti-tumor battle (Video S1).

### Universality of the Solution

**Figure 3:**
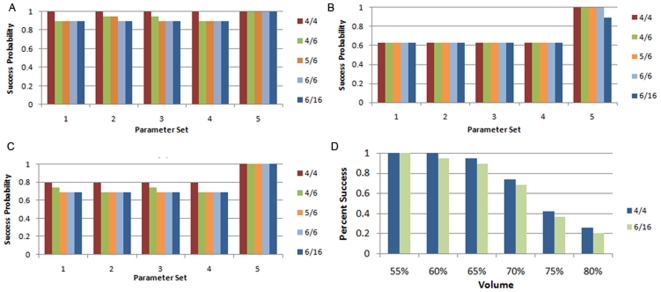
Challenging the rescue protocol with more aggressive tumors. Increased tumor resistance to apoptosis is defined by increasing the threshold representing the number of signals required to induce apoptosis in both tumor and normal cells (written as tumor threshold/normal threshold in graph legend). Protocol success rates (y-axis) are presented for increasing proliferation ratios [(a) 6, (b) 10, (c) 20, as described in the text] for 5 different sets of rescue protocol parameters seen on the x-axis [“I'm dying” signals (radius, strength), and “please die” signals (radius, strength), as described in Table 1]. Since experiments are stochastic, 100% success denotes that all experiments with the same protocol parameters succeeded in removing all tumor cells without significantly harming the healthy tissue. Each graph represents data from 570 experiments demonstrating that success rates are high for a large range of parameters, between 62 and 100%. This shows robustness of the protocols for biological heterogeneity. A range of threshold values are shown (graph legend) to demonstrate that even with a significant increase in number of signals necessary for tumors to die, the protocols still have very similar success rates to the lower thresholds. This is counterintuitive, and shows robustness of the protocols to tumor resilience and thus supports the correctness of the proposed mechanism. (d) When the cancer volume increases before a delayed initialization of the rescue protocols, complete eradication of cancer cells can still be possible. As the initial volume increases, the success rate decreases; however, we can still eradicate tumors for a high percentage of beginning tumor volumes. This signifies that our proposed signaling mechanisms can facilitate tumor eradication even if they start late.

To confirm the robustness of the combined-protocol solution, we tested the simulation with numerous parameter ranges, and introduced tissue heterogeneity. The following were introduced: varying tumor proliferation ratios, modifying external apoptosis request parameters (signal strength, radius), tissue heterogeneity for proliferation parameters in normal and tumor cells, changing initial tumor volume, and increasing tumor cells' resistance to external apoptosis commands. The latter was viewed by both deterministic and stochastic methods, which relate to resistance at the system or the cell level, respectively. For the deterministic method, resistance was defined by increasing the number of requests required for tumor cells to comply with external apoptotic requests. The results of several thousand computational runs are presented in Figures 3a–c, showing high success rates which vary between 62 and 100%. We interpret the high 100% of success as correlating to a high potential of tumor removal in a natural biological system. The higher response thresholds (6/6 and 6/16) tested for parameter sets 4 and 5 lead to the same success rate as the majority of lower thresholds despite cells needing to receive significantly more signals to comply. This suggests that in most cases variation in resistance to external apoptotic commands does not have a substantial effect on rescue protocol success rates. As proliferation ratios increase fewer experiments succeed; however, the protocols are still able to remove all tumors in the majority of the experiments. Not all rescue protocol parameters necessarily give complete success, but there still exist choices for each proliferation ratio that can lead to a majority of success in removing tumor cells from the system.

**Table 1:** Rescue Protocol Parameters.

Label	Tumor I'm Dying	Normal I'm Dying	Normal Please Die
1	1/1	1/1	1/1
2	1/2	1/2	1/1
3	1/2	1/2	1/2
4	1/3	1/3	1/3
5	2/3	1/2	1/2

Each column shows radius/strength for the specified signal type. Radius represents how far the signal propagates in cellular space units, strength shows the starting value of the amount of signal observed by the receiving cells which decreases over the given distance, and the label is for easy reference to a set.

For results represented in Figures 3a–c, it was assumed that the rescue protocols start with an early recognition of cancer cells. However, we also tested how the protocols would function if they are held inactive until a particular volume of tumor cells was reached (Figure 3d). The proposed protocols were still successful even with up to 60% of the system occupied by tumor cells before their activation. These results indicate a window of opportunity for re-activation of inactive rescue protocols in our model in which they can still successfully eradicate the tumors. Success rates dropped significantly with initiation volumes above 60% (Figure 3d).

**Figure 4:**
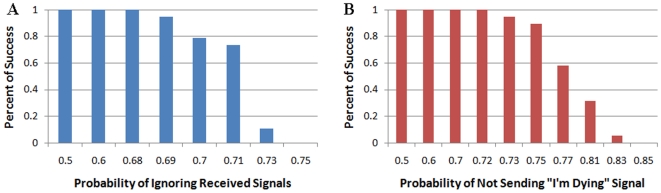
Stochastic presentation of tumor resistance to apoptosis induction. The rescue protocols are robust, as summarized from 665 random sampled experiments. (a) A system where cancer cells ignore up to 68% of signals still results in 100% success. (b) A system where cancer cells do not send I'm Dying signals up to 72% of the time still results in 100% success. These results show that we do not necessarily need to assume that tumor cells will follow the rescue protocols consistently, but that they can fail the majority of the time and still result in complete eradication of tumor cells in the system. Only very high rates of failure complying with the rescue protocols within the cancer cells will result in a loss of tissue integrity. We see a sharp decrease in success rates once the protocols reach that high failure level, demonstrating that once the point of protocol break is crossed, any greater break will cause the system to deteriorate even faster.

For consideration of tumor resistance by stochastic analysis we introduced parameters related to cancer cells ignoring some external apoptotic signals (Figure 4a) or failing to emit some of the “I'm dying” reports (Figure 4b). Success rates above 55% were measured for tumor cells ignoring up to 71% of received signals or failing to emit up to 77% of “I'm dying” messages. This result implies that the suggested protocols are so robust that they will succeed even when the majority of their signals do not function properly and many of the cells do not adhere to them. Only a minority of cells adhering to the protocols is sufficient for saving the system from tumor cells.

## Discussion

This paper introduces a functional mathematical model where algorithms provide a framework to describe biological events in an active society of cycling cells. Biological occurrences are conceived as propagation of information units, cell cycle events are described as “basic life rules,” and extra-cellular events are presented as “communication protocols.” A phenotypic description requires that normal cells use cell cycle check points to correct major damage or induce self death, and cancer cells display inappropriate intrinsic apoptosis signaling and relative resistance to external apoptosis commands, ignorance of spatial regulations, and advantageous proliferation. Curing in our system requires total cancer cell disappearance and healthy system recovery, all specified in the anti-cancer battle time frame. To the best of our knowledge this is the first mathematical model to accommodate all of these considerations, and to provide innovative solutions based on Artificial Intelligence approaches. The main logical solution raised by the model is that the optimal mechanism to induce selective tumor apoptosis requires the acknowledgment and utilization of active “citizenship properties” of each cell, normal or tumor, to its best availability. This mechanism is based on “rescue communication protocols” where cells report their viability status and emit and respond to external apoptotic requests, defined as “please die” and “I'm dying” signals. It is supported by tens of thousands of data runs challenging the accommodation potential of the system and the robustness of the identified logical solution for various cancer conditions, all of which have met with success. The solution can be viewed philosophically as a shared responsibility for system health: each cell attempts to maintain its own health, and when major cellular damage occurs the responsibility is shared by the society. The solution is limited, however, by increasing initial tumor volume (above 60% in our model) and elevated number of tumor cells (above 71–77%) with absolute resistance to communication within the system.

The role of the tumor microenvironment in cancer development and progression as well as in clinical outcome is increasingly acknowledged; furthermore it can now be viewed at the genomic level (summarized in [26]). Specifically for the tumor-environment interaction suggested in our model, there are emerging data to support the significance of cancer cells reporting their death. In vitro models further supported by clinical outcome data showed that HMBOX1 release by tumor cells exposed to chemotherapy is essential in mediating effective tumor eradication by different chemotherapy agents and solid tumor models; HMBOX1 release functions to report initial cell damage by chemotherapy to the immune system and likely cooperates with additional messengers released by dying cells as its artificial introduction does not lead to the same effect [16], [18]. The computerized “I'm dying” signal likely parallels this biological mechanism.

The clinical significance of external apoptosis stimulation in inducing selective tumor cell death is supported by early clinical trials with the recombinant human apoptosis ligand 2/Tumor Necrosis Factor-Related Apoptosis-Inducing Ligand as reviewed by Ashkenazi et al [19]. Our artificial model results suggest that optimal selective cancer cell eradication requires recognition of cancer cell death messages, activation of the external apoptotic pathway, and some residual compliance with apoptotic induction to be coordinated. It is beyond the scope of this paper, however, to test and prove biological and clinical co-equivalents to our computational results.

There are several limitations to our work. First, findings, though demonstrated by numerous system experiments, should be viewed with the limitation of a simplified model and in light of the basic assumptions delineated in the introduction section. Still, as with all models, it is this simplification that allows the establishment of basic concepts essential for identification of fundamental elements in complex biological systems. The model is currently mature enough to incorporate multiple biological data (as demonstrated in the tissue heterogeneity experiments) and in its next generation, now under development, will incorporate the effects of angiogenesis and anti-cancer chemotherapy agents. Second, mechanisms and experiments are all viewed by functionality, and require back translation to proteins and genes with molecular proof. While discussing biological and possible clinical co-equivalents, additional work is necessary to determine the molecular and clinical fit to our system solutions.

In conclusion, the system is built to test and identify solutions beyond time series predictions and therefore give substantially more than an analytical study of the data input. Our system can be used conceptually for screening and comparing biological experiments with only a minimal cost, and enables storage of configurations with applications tested from any desired point. It has determined specific “rescue protocols” that may be crucial for selective apoptosis of tumor cells, which are activated naturally and may be activated by external triggering as well. This model simplifies the complexity of cancer occurrence, and when combined with data driven techniques has the potential to facilitate a novel step forward in recognizing optimized anti-cancer interventions.

## Supporting Information

Video S1 Simulation Movie. Growth of normal cells in the model, followed by tumor growth when there are no rescue signals, and then tumor growth with both rescue protocols. Time is sped up from original simulation, but no other alterations have been made.
(1.83 MB MOV)
